# Exercise-Induced Intraventricular Gradients As a Potential Cause of Sudden Cardiac Death

**DOI:** 10.7759/cureus.41408

**Published:** 2023-07-05

**Authors:** Carlos A Cotrim, Nuno Cotrim, Jorge H Guardado, Luis Baquero

**Affiliations:** 1 Heart Center, Hospital da Cruz Vermelha, Lisboa, PRT; 2 Cardiology, Unidade Cardiovascular de Riachos, Riachos, PRT; 3 Cardiology, Hospital Particular do Algarve, Faro, PRT; 4 Cardiology, Hospital de Santarém, Santarém, PRT

**Keywords:** sudden cardiac death, children adolescents, sports cardiology, intraventricular gradients, exercise stress echocardiography

## Abstract

A 16-year-old boy reported an episode of dizziness related to intense training six months before an episode of aborted sudden death. The screening required for competitive sports practice was normal. There were no personal or familial antecedents of sudden death or heart disease. After winning a triathlon competition, he experienced a cardiac arrest episode. He received defibrillation with the return of spontaneous circulation. A medical evaluation that included electrocardiogram (ECG) and echocardiogram had normal results. A complete study including cardiac MRI, coronary CT angiography, a genetic study for heart disease, the flecainide test, and a stress echocardiogram with ergometrine was done, and all results were normal. During a Holter ECG and exercise stress echo, isolated premature ventricular complexes were detected. During the effort treadmill stress echocardiogram, the athlete developed a significant intraventricular obstruction with an end-systolic peak, without systolic anterior movement of the mitral valve, which disappeared in the first minute of the recovery. We highlight the possible cause-effect relation between the events.

## Introduction

The development of significant intraventricular gradients during exercise is rare, and it is usually associated with left ventricular hypertrophy [1]. We have previously detected significant intraventricular gradient and systolic anterior movement of the mitral valve in athletes who passed screening for sports practice and had structurally normal hearts [2]. In that study, the prevalence of intraventricular gradients associated with exercise in athletes with symptoms was frequent. The method used in this research had the particularity of performing the echocardiographic evaluation during recovery in the orthostatic position which leads to a reduction in venous return and preload resembling what athletes experience during daily practice. 

We describe a triathlon athlete with the same phenomenon detected in medical evaluation after aborted sudden cardiac death. A possible role of exercise stress echocardiography for intraventricular pressure gradient search in athletes with normal hearts is discussed.

This article was previously presented as a meeting abstract at the 2019 Euroecho in Vienna.

## Case presentation

**Figure 1:**
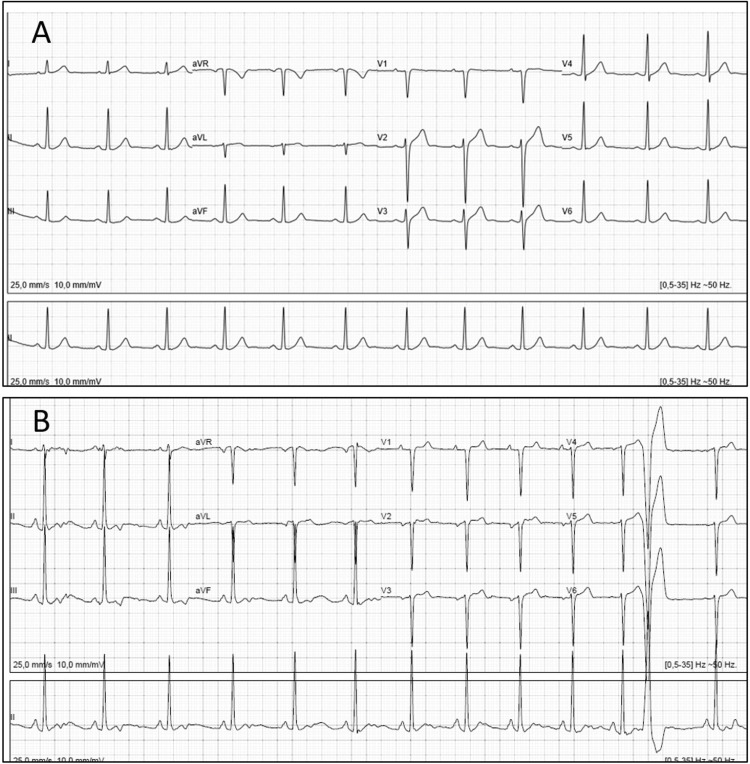
ECG 12 months after stopping competitive exercise (A) before and (B) at the end of exercise stress echocardiogram

A 16-year-old boy had an episode of dizziness in relation to intense exercise six months before an episode of aborted sudden death. The evaluation required for sports practice including clinical history, complete physical evaluation, and an electrocardiogram (ECG) had yielded normal results, and there was no familial history of sudden death or heart disease. 30 minutes after winning a triathlon competition, the athlete had a cardiac arrest episode. He presented with ventricular fibrillation and was defibrillated with the return of spontaneous circulation. A complete medical evaluation yielded normal results. The ECG and echocardiogram were normal. A complete study including cardiac MRI, coronary CT angiography, complete genetic study for heart disease, flecainide test, and stress echocardiogram with ergometrine, were done, and all tests had normal results. A 24-hour Holter and exercise stress echo revealed frequent isolated premature ventricular complexes (Figure 1).

 

**Figure 2:**
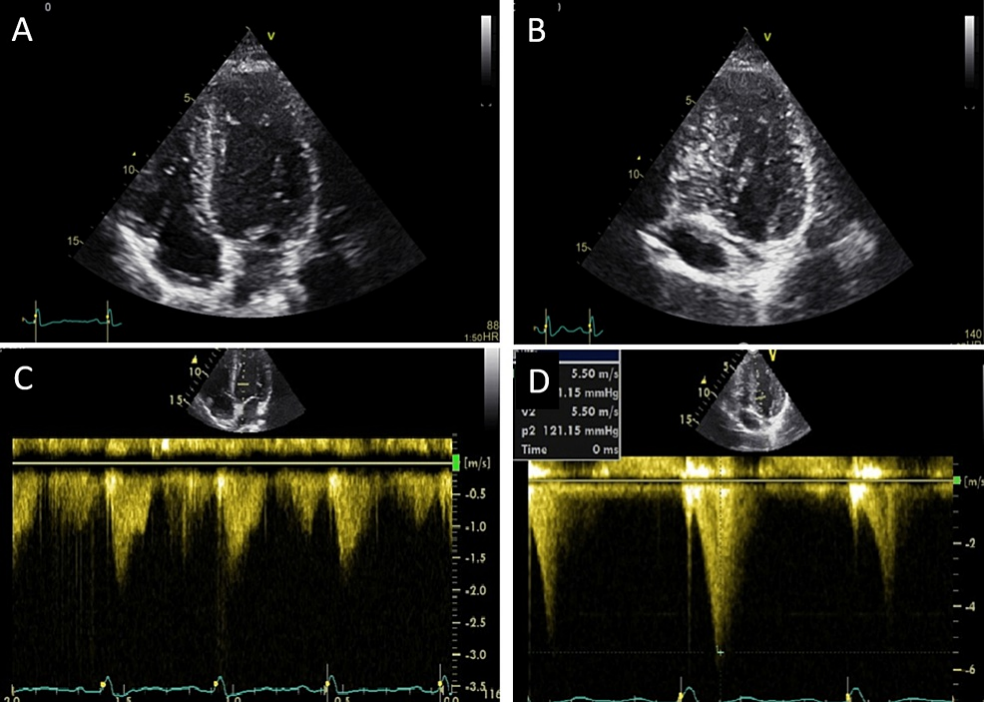
(A) Apical four-chamber before exercise; (B) Apical four-chamber at peak exercise; (C) CW Doppler at rest without significant intraventricular gradient D and at peak exercise with significant intraventricular gradient CW: Continuous Wave

During the treadmill exercise stress echocardiogram, a significant intraventricular obstruction with an end-systolic peak, without systolic anterior motion was detected (Figure 2), which disappeared within the first 60 seconds after exercise cessation.

The patient underwent stress echo without a fall in blood pressure and the study was negative for myocardial ischemia. The athlete refused implantable cardioverter defibrillator (ICD) implantation and started bisoprolol 2.5 mg daily. Informed consent for publication was obtained from the athlete.

## Discussion

Small intraventricular gradients exist during normal cardiac function. These gradients increase during exercise and may be explained by three possible mechanisms: augmentation of nonobstructive physiological gradients; obstruction at the end of systole related to ventricular cavity obliteration, which seemed to have occurred in the athlete in the current case; or obstruction at mid-systole caused by the systolic anterior motion of the mitral valve, limiting ejection [3]. When the systolic anterior motion of the mitral valve occurs, the geometry of the left ventricle chamber or the mitral valve apparatus as an elongated mitral leaflet and anterior displacement of papillary muscles or increase in relative wall thickness. Such changes were not visualized in our patient, although maneuvers that change loading conditions in structurally normal hearts have been demonstrated to be able to cause intraventricular obstruction [4]. Exercising and participating in sports could provoke these changes in loading conditions [2]. 

The phenomenon of sudden death in young athletes has been studied, and the most frequent causes are agreed to be hereditary or congenital [5]. However, in a review of causes of sudden deaths during sports activities, around 30% of anatomopathological studies showed no abnormalities [6]. This finding suggests that screening programs which assess normal electrical and mechanical function of the heart are failing to detect and prevent the true cause of sudden cardiac death. In the current case, the patient had a normal echocardiogram, normal coronary arteries, and normal MRI, which means that a morphological study of the patient’s heart would likely reveal no abnormalities. The intraventricular gradients detected during and immediately after exercise could well have been responsible for ischemia leading to arrhythmias, and this hypothesis must be considered as possible [7]. The medical examination of this athlete was conducted because of an aborted sudden cardiac arrest that occurred after intense effort. Although we did not reproduce the event during the exercise test, we detected an anomaly in cardiac function that may have caused the cardiac arrest episode to occur [2]. This abnormality was only detectable during and immediately after effort, and it is not listed among the conditions that contraindicate participation in competitive sports, according to recommendations of the European Society of Cardiology [8]. We believe that intraventricular gradients induced by exercise as accepted cause of myocardial ischemia could be among the etiologies that cause sudden death in cases in which anatomopathological studies reveal no abnormalities, and we accordingly disqualified the athlete from sports practice, prescribed bisoprolol 2.5 mg once daily, and proposed the implantation of an ICD [9]. The athlete refused device implantation.

We do not agree with other groups that suggest that intraventricular gradients are a normal finding even in symptomatic children without hypertrophic cardiomyopathy [10]. We underline that this finding has been almost excluded as a normal response to exercise in young healthy adults [11]. In addition, the present case and our previous experience with a significant number of athletes reinforce this notion [2].

In our clinical practice, beta blockers are the mainstay of therapy when intraventricular gradients and certain arrhythmias are detected during exercise echocardiography [2,12]. Later, the exam is repeated to ascertain the efficacy of the implemented therapy. If the patient becomes asymptomatic and does not develop significant arrhythmias nor intraventricular gradients, the athlete and family decide to proceed or not with the sport's practice. 

## Conclusions

In the case described, significant abnormalities in cardiac function were found only during and after exercise. This outcome reinforces that this methodology should be used in athletes who have symptoms or aborted sudden death related to exercise but do not have structural abnormalities of the heart. We suggest that athletes with symptoms who develop significant intraventricular gradients during exercise should undergo careful follow-up to enable a more accurate assessment of their clinical significance.

## References

[1] Peteiro J, Montserrat L, Castro-Beiras A (1999). Labil subaortic obstruction during exercise stress echocardiography. Am J Cardiol.

[2] Cotrim C, Almeida AR, Miranda R, Almeida AG, Cotrim H, Picano E, Carrageta M (2010). Stress-induced intraventricular gradients in symptomatic athletes during upright exercise continuous wave Doppler echocardiography. Am J Cardiol.

[3] Yotti R (2004). ¿Qué significado tiene un gradiente de presión intraventricular sistólico durante el ejercicio? (Article in Spanish). Rev Esp Cardiol.

[4] Grose R, Maskin C, Spindola-Franco H, Yipintsoi T (1981). Production of left ventricular cavitary obliteration in normal man. Circulation.

[5] Mont L, Pelliccia A, Sharma S (2017). Pre-participation cardiovascular evaluation for athletic participants to prevent sudden death: position paper from the EHRA and the EACPR, branches of the ESC. Endorsed by APHRS, HRS, and SOLAECE. Europace.

[6] Suárez-Mier MP, Aguilera B (2002). Causas de muerte súbita asociada al deporte en España (Article in Spanish). Rev Esp Cardiol.

[7] Galiuto L, Picano E (2015). Stress echo in microvascular disease. Stress Echocardiography.

[8] Lancellotti P, Pellikka PA, Budts W (2017). The clinical use of stress echocardiography in non-ischaemic heart disease: recommendations from the European Association of Cardiovascular Imaging and the American Society of Echocardiography. J Am Soc Echocardiogr.

[9] Cotrim C, Almeida AG, Carrageta M (2008). Exercise-induced intra-ventricular gradients as a frequent potential cause of myocardial ischemia in cardiac syndrome X patients. Cardiovasc Ultrasound.

[10] Wittlieb-Weber CA, Cohen MS, McBride MG (2013). Elevated left ventricular outflow tract velocities on exercise stress echocardiography may be a normal physiologic response in healthy youth. J Am Soc Echocardiogr.

[11] Cabrera Bueno F, Rodríguez-Bailón I, López-Salguero R, Gómez-Doblas JJ, García-Pinilla JM, de Teresa Galván E (2006). Efecto del ejercicio sobre las velocidades de flujo sistólico ventricular izquierdo en adultos sanos (Article in Spanish). Rev Esp Cardiol.

[12] Cotrim C, Lopes LR, Almeida AR (2010). Efficacy of beta-blocker therapy in symptomatic athletes with exercise-induced intra-ventricular gradients. Cardiovasc Ultrasound.

